# Opposite Macrophage Polarization in Different Subsets of Ovarian Cancer: Observation from a Pilot Study

**DOI:** 10.3390/cells9020305

**Published:** 2020-01-27

**Authors:** Ann Vankerckhoven, Roxanne Wouters, Thomas Mathivet, Jolien Ceusters, Thaïs Baert, Anaïs Van Hoylandt, Holger Gerhardt, Ignace Vergote, An Coosemans

**Affiliations:** 1 ImmunOvar Research Group, Laboratory of Tumor Immunology and Immunotherapy, Department of Oncology, KU Leuven, 3000 Leuven, Belgiumroxanne.wouters@kuleuven.be (R.W.); jolien.ceusters@kuleuven.be (J.C.); thais.baert@gmail.com (T.B.); anais.vanhoylandt@uzleuven.be (A.V.H.); ignace.vergote@uzleuven.be (I.V.); 2 Oncoinvent AS, 0484 Oslo, Norway; 3 PARCC, HEGP Institute (team 9), INSERM U970, Université Paris Descartes, 75006 Paris, France; thomas.mathivet@gmail.com; 4 Vascular Patterning Lab, Center for Cancer Biology, VIB, KU Leuven, 3000 Leuven, Belgium; holger.gerhardt@kuleuven.vib.be; 5 Department of Gynecology and Gynecologic Oncology, Kliniken Essen Mitte (KEM), 45136 Essen, Germany; 6 Department of Gynecology and Obstetrics, Leuven Cancer Institute, University Hospitals Leuven (UZ Leuven), 3000 Leuven, Belgium; 7 Laboratory of Gynecologic Oncology, Department of Oncology, KU, Leuven, 3000 Leuven, Belgium

**Keywords:** ovarian cancer, macrophages, M2

## Abstract

The role of the innate immune system in ovarian cancer is gaining importance. The relevance of tumor-associated macrophages (TAM) is insufficiently understood. In this pilot project, comprising the immunofluorescent staining of 30 biopsies taken from 24 patients with ovarian cancer, we evaluated the presence of total TAM (cluster of differentiation (CD) 68 expression), M1 (major histocompatibility complex (MHC) II expression), and M2 (anti-mannose receptor C type 1 (MRC1) expression), and the blood vessel diameter. We observed a high M1/M2 ratio in low-grade ovarian cancer compared to high-grade tumors, more total TAM and M2 in metastatic biopsies, and a further increase in total TAM and M2 at interval debulking, without beneficial effects of bevacizumab. The blood vessel diameter was indicative for M2 tumor infiltration (Spearman correlation coefficient of 0.65). These data mainly reveal an immune beneficial environment in low-grade ovarian cancer in contrast to high-grade serous ovarian cancer, where immune suppression is not altered by neoadjuvant therapy.

## 1. Introduction

Ovarian cancer has the fifth highest mortality rate among women diagnosed with cancer in Europe [1]. Based on its histopathologic and genetic profile, epithelial ovarian cancer (EOC) can be subdivided into different subtypes: endometrioid, clear-cell, mucinous, and serous carcinomas [2]. Serous ovarian cancer is the most common subtype, accounting for ±75% of all EOC, and it can be further subdivided into low-grade and high-grade serous ovarian cancer [3]. Due to the absence of distinct symptoms, the majority is diagnosed at an advanced stage disease (International Federation of Gynecology and Obstetrics (FIGO) stage III (51%) or stage IV (29%)) [4]. The backbone of first-line treatment consists of a primary debulking surgery (PDS) and platinum-based chemotherapy. However, patients that are not eligible for PDS can instead receive neoadjuvant chemotherapy (NACT) followed by interval debulking surgery (IDS) [5,6]. Despite this extended first-line treatment, the majority of patients relapse. Overall, patients in an advanced disease stage have a poor five-year survival of only 25% [4,7].

The immune system has an important role in ovarian cancer initiation, progression, and prognosis. However, most data in this regard exist on the adaptive immune system [8,9]. Nevertheless, our group recently provided evidence on the importance of the innate immune system in ovarian cancer [10,11]. The major players within this innate immune system are dendritic cells, myeloid derived suppressor cells, and macrophages. Macrophages infiltrating the tumor microenvironment (TME) are defined as tumor-associated macrophages (TAMs). Although they are known to have a high degree of plasticity and heterogeneity, the majority of macrophages can be classified into two phenotypes based on their activation status. The classically activated macrophages (M1, type I) express a broad series of proinflammatory and immunostimulatory effector molecules, such as interleukin-1-beta and tumor necrosis factor alpha, and they possess a strong anti-tumoral activity. This is in contrast to the alternatively activated macrophages (M2, type II) which are characterized by a tumor promoting phenotype, as well as expression a wide array of anti-inflammatory effector molecules such as interleukin 10 and tumor necrosis factor beta, thereby contributing to a more immunosuppressive TME [12]. However, very recent data showed that the gene expression profile between tumor-resident macrophages and TAM is different and that TAM cannot always be dichotomized into a pure M1 or pure M2 phenotype [13].

Previous studies already demonstrated the importance of TAMs in ovarian cancer. TAMs in the ascites of ovarian cancer patients were shown to express B7-H4+, resulting in a dysfunctional phenotype and suppressing T-cell anti-tumor immunity [14]. A study by Zhang et al. (2014) demonstrated that an increased M1/M2 ratio correlated with improved prognosis in ovarian cancer patients [15], indicating the importance of this balance between immune stimulatory M1 and tumor-promoting M2 macrophages. 

In addition to the relevance of the immune system in ovarian cancer development, angiogenesis is another important hallmark [16]. Angiogenesis is defined as the expansion of vascular endothelial cells and the development of new blood vessels in order to provide sufficient supply of essential nutrients such as oxygen. As tumor cells are rapidly dividing, they have an increased need for nutrients and, therefore, they induce angiogenesis by secreting various growth factors such as vascular endothelial growth factor (VEGF) [17]. However, the newly formed vasculature network in tumors is highly irregular and often comprises leaky blood vessels [17,18]. This dysfunctional vascularization results in chronic hypoxia and contributes to metabolic changes in tumor cells, making them more resistant to therapy. Moreover, they facilitate the dissemination of cancer cells to metastatic sites [19]. In ovarian cancer, bevacizumab (anti-VEGF receptor 2 (VEGFR2) monoclonal antibody) is one of the few targeted therapies available for patients, inhibiting the binding of VEGF to its receptor and, therefore, regulating and normalizing the blood vessels [20,21]. However, VEGF is a factor produced by a variety of cells, including macrophages. The presence of VEGF stimulates both the migration of macrophages to the TME and their polarization toward a more M2 phenotype [17]. Furthermore, macrophages were shown to facilitate resistance to anti-VEGF treatment [22].

In this exploratory pilot study, we evaluated different ovarian cancer tumor biopsies for the presence of total TAM (cluster of differentiation (CD) 68 expression), M1 (major histocompatibility complex (MHC) II expression), and M2 (anti-mannose receptor C type 1 (MRC1) expression), and for the blood vessel diameter. Our results highlight differences between low-grade and high-grade tumor, as well as between metastases and primary tumor biopsies, and they underscore the relationship between the blood vessel diameter and macrophage infiltration. We also explored if platin-based chemotherapy and bevacizumab altered the macrophage composition.

## 2. Materials and Methods

### 2.1. Patients and Clinical Data 

We prospectively collected 30 tissue samples from 24 patients diagnosed with ovarian cancer at the University Hospitals Leuven (Belgium) in the OV-IMM-2014 study. Patients with known immune diseases or taking immunosuppressive drugs at diagnosis, infectious disease (human immunodeficiency virus (HIV), hepatitis B and C), or concurrent non-ovarian cancer at the moment of diagnosis were excluded from the study. Samples were collected between June 2014 and April 2016. We collected tumor samples at diagnosis (treatment-naïve, PDS) and/or during IDS (after chemotherapy and/or bevacizumab treatment). The study was approved by the ethical committee of the University Hospitals Leuven (s56311). All patients included gave written informed consent.

### 2.2. Immunofluorescence Staining

Tumor biopsies were fixed with 4% paraformaldehyde for 24 h, washed with DPBS (Dulbecco’s phosphate-buffered saline), and stored at 4 °C until further processing. Samples were prepared as 200-µm-thick vibratome sections, blocked and permeabilized in TNBT buffer (0.1 M Tris pH 7.4; NaCl 150 mM 0.5% blocking reagent from Perkin Elmer (Waltham, Massachusetts, USA), 0.5% Triton X-100) for four hours at room temperature. Tissues were incubated overnight at 4 °C with the following primary antibodies diluted in TNBT buffer: anti-glucose transporter-1 (Glut1) (Millipore, Burlington, Massachusetts, USA; 1:200 dilution), anti-CD68 (Abcam, Cambridge, UK; 1:200 dilution), anti-MHC-II (Thermo Scientific, Waltham, Massachusetts, USA; 1:100 dilution), or anti-MRC1 (R&D Systems, Minneapolis, Minnesota, USA; 2 ug/mL). Next, slides were washed in TNBT buffer and incubated overnight at 4 °C with the appropriate secondary antibody coupled with Alexa 488/555 (Life Technologies, Carlsbad, California, USA; 1:200 dilution) diluted in TNBT buffer. Tissues were washed and mounted on slides in fluorescent mounting medium (Dako/Agilent, Santa Clara, California, USA). Images were acquired using a Leica TCS SP8 confocal microscope. Semi-automated quantification analyses were performed using Fiji software [23] to assess the number of CD68^+^, MHCII^+^, and MRC1^+^ cells per field. Five fields were assessed per sample, and the average was used in all subsequent analyzes.

### 2.3. Statistical Analyses

The expression of three biomarkers (CD68, MHCII, MRC1) and the blood vessel width (blood vessels visualized by Glut1 staining) were compared between FIGO stages (FIGO stage I, FIGO stage III, and FIGO stage IV), bioptic sites (primary tumor and metastatic tumor), tumor grades (low-grade ovarian carcinoma and high-grade ovarian carcinoma), moment of surgery (PDS and IDS), and administered therapy (chemotherapy and chemotherapy + bevacizumab). For this, we visualized the data with box plots. Spearman’s rank-order correlation was used to correlate MRC1 with Glut1. We performed this exploratory analysis using R version 3.5.1.

## 3. Results

### 3.1. Description of Patient Samples

Tumor biopsies were collected for 24 patients. Patient characteristics are displayed in Table 1. 

### 3.2. TAMs at Ovarian Cancer Diagnosis

**Figure 1:**
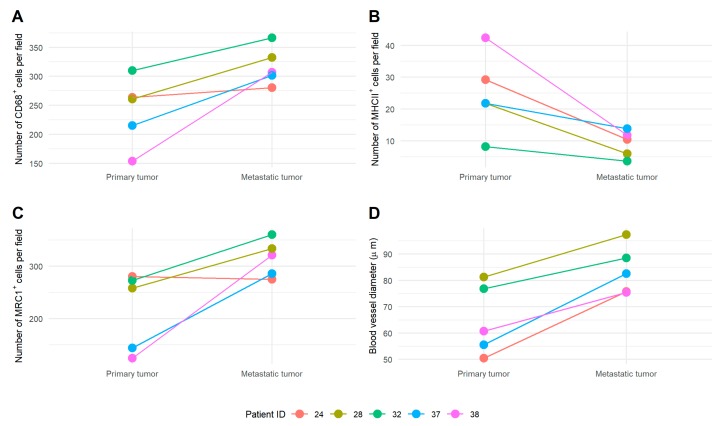
Comparison of macrophage profile and blood vessel diameter in matched biopsies at primary and metastatic tumor sites. The amount of (**A**) cluster of differentiation (CD) 68^+^, (**B**) major histocompatibility complex (MHC) II^+^, and (**C**) anti-mannose receptor C type 1 (MRC1)^+^ cells for five patients with matched biopsies, and (**D**) blood vessel diameter (µm) based on glucose transporter-1 (Glut1) expression. Samples of patients 37 and 38 were collected at primary debulking surgery (PDS), while samples of patients 24, 28, and 32 were collected at interval debulking surgery (IDS).

**Figure 2:**
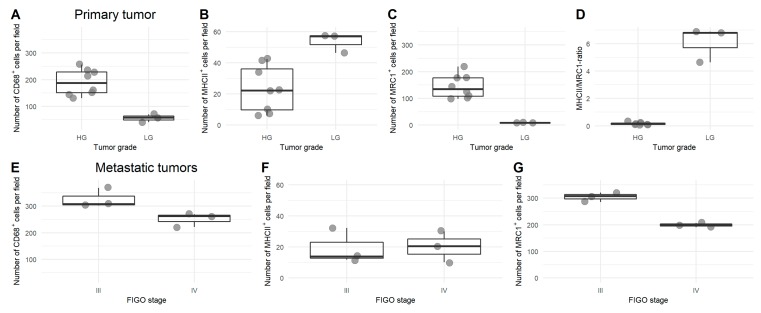
Comparison of macrophage profile in low-grade versus high-grade tumors and in International Federation of Gynecology and Obstetrics (FIGO) stage III versus IV. (**A**–**C**) Number of CD68^+^ (**A**), MHCII^+^ (**B**), and MRC1^+^ (**C**) cells per field in primary high-grade and low-grade tumor samples. Ratio between MHCII^+^/MRC1^+^ (i.e., M1/M2) in high-grade vs. low-grade ovarian cancer (**D**). (**E**–**G**) Number of CD68^+^ (**D**), MHCII^+^ (**E**), and MRC1^+^ (**F**) cells per field in metastatic biopsies of stage III and stage IV ovarian cancer.

**Figure 3:**
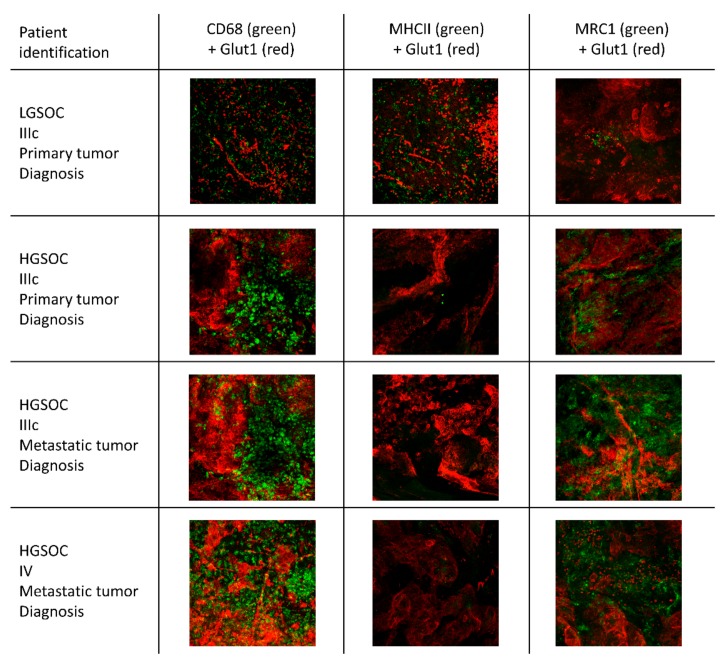
Comparison of macrophage infiltration between different subsets of ovarian cancer. Immunofluorescence staining for CD68, MHCII, MRC1, and Glut1 in low-grade serous ovarian cancer (LGSOC) and high-grade serous ovarian cancer (HGSOC), in both primary and metastatic tumors at diagnosis. Each image is 500 µm × 500 µm.

At diagnosis, metastatic tumor sites showed more total TAMs and more M2 TAMs compared to the primary tumor. This was true for a comparison in bulk, but also for a paired sample comparison (Figure 1 and Figure S1, Supplementary Materials). Low-grade ovarian cancer showed less total TAMs, more M1 TAMs, and less M2 TAMs compared to high-grade ovarian cancer at diagnosis in the primary tumor (no metastatic biopsies available of low-grade tumors). M2 TAMs were less abundant in stage IV ovarian cancer, compared to stage III in metastatic tumors, while there were no changes in primary tumors (Figure 2). Representative fluorescent pictures are shown in Figure 3. 

### 3.3. Effect of Platin-Based Chemotherapy and Bevacizumab on the Presence of TAM in High-Grade Serous Ovarian Cancer Biopsies

**Figure 4:**
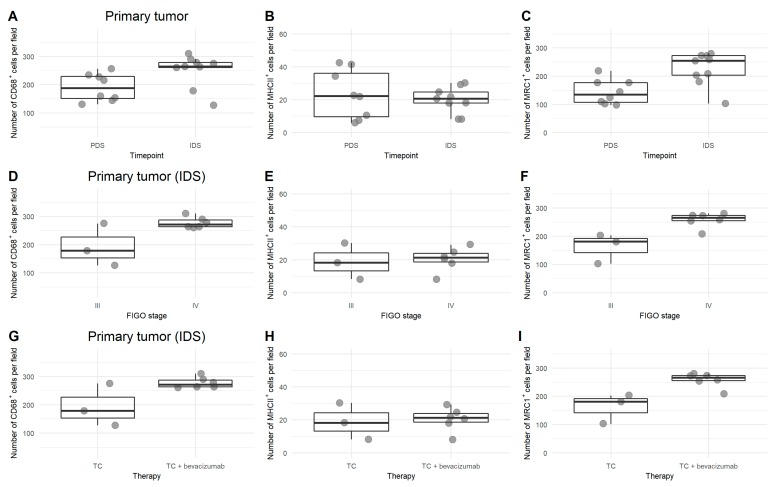
Comparison of macrophage profile after platin-based chemotherapy and bevacizumab in HGSOC. (**A**–**C**) Number of CD68^+^ (**A**), MHCII^+^ (**B**), and MRC1^+^ (**C**) cells per field in primary tumor samples at diagnosis (PDS = primary debulking surgery and at IDS = interval debulking surgery, i.e., after receiving platin-based chemotherapy). (**D**–**F**) Number of CD68^+^ (**D**), MHCII^+^ (**E**), and MRC1^+^ (**F**) cells per field in stage III vs. stage IV ovarian cancer after platin-based chemotherapy. (**G**–**I**) Number of CD68^+^ (**G**), MHCII^+^ (**H**), and MRC1^+^ (**I**) cells per field in primary tumor samples at IDS, after receiving platin-based chemotherapy alone or in combination with bevacizumab.

Platin-based chemotherapy increased the amount of total and M2 macrophages in HGSOC. This was most pronounced in biopsies of the primary tumor (Figure 4A–C) and in stage IV ovarian cancer patients (Figure 4D–F). The additional use of bevacizumab compared to paclitaxel-carboplatin alone increased the number of total TAMs and M2 when evaluated in interval debulking samples at the primary tumor site (Figure 4G–I).

### 3.4. Blood Vessel Abnormality in Ovarian Cancer and Its Relationship with M2 Macrophages

**Figure 5:**
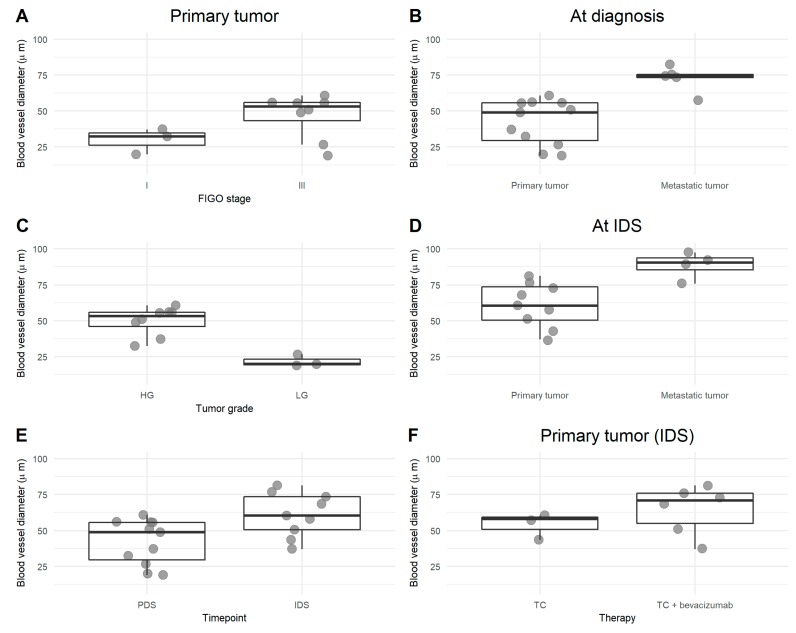
Comparison of blood vessel diameter (µm). Blood vessels were stained for Glut1. Diameter of the visualized blood vessels was measured subsequently, and results are presented on the *Y*-axis. Comparison of primary tumor samples of early vs. late stage ovarian cancer (**A**), of primary vs. metastatic tumor samples at diagnosis (**B**), of high-grade (HG) versus low-grade (LG) tumors (**C**), after platin-based chemotherapy (**D**,**E**), and after the addition of bevacizumab (**F**).

**Figure 6:**
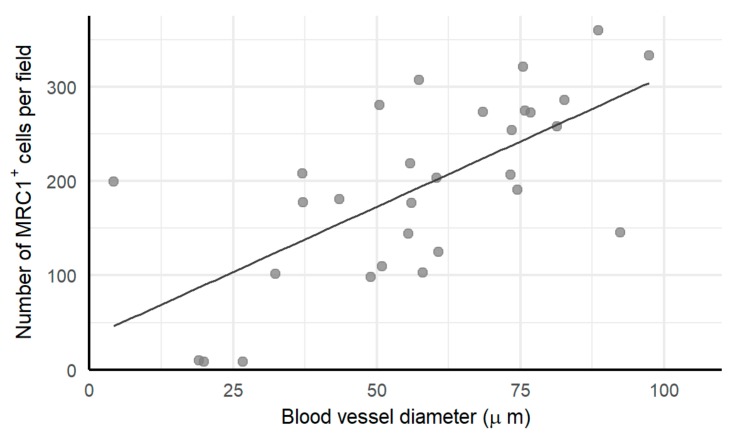
Correlation between blood vessel width and M2 macrophages. A larger blood vessel denotes more M2 macrophages infiltrating the tumor (Spearman correlation coefficient of 0.65).

**Figure 7:**
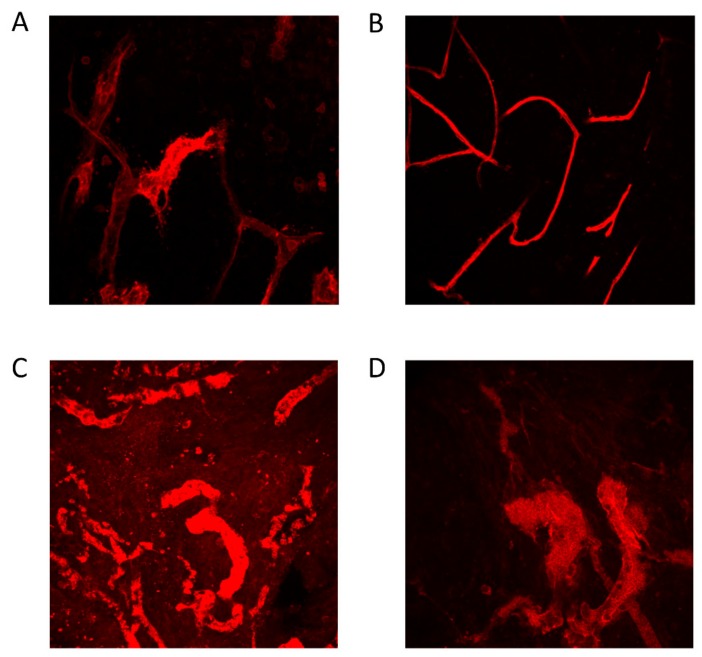
Comparison in blood vessel width (Glut1) between high-grade serous ovarian cancer (HGSOC) and low-grade serous ovarian cancer (LGSOC) (**A**,**B**) and between primary and metastatic tumor (**C**,**D**). (a) Primary tumor biopsy of a stage IIIC HGSOC. (**B**) Primary tumor biopsy of a stage IIIC LGSOC. (**C**) Primary tumor biopsy of HGSOC stage IIIC (different patient than (**A**)). (**D**) Metastatic tumor biopsy of the same patient as (**C**). Each image is 500 µm × 500 µm.

Blood vessel diameter increased with increasing stage and grade and was also larger in metastatic tumors compared to primary tumors, both at diagnosis and at interval debulking. Chemotherapy seemed to increase the blood vessel width, as did bevacizumab (Figure 5). Wider blood vessels resulted in more M2 macrophages (Figure 6). Representative pictures are presented in Figure 7.

## 4. Discussion 

This exploratory pilot experiment aimed to visualize the presence of TAMs in different subsets of ovarian cancer. At diagnosis, we found that the M1/M2 ratio was high in low-grade ovarian cancer and that, in metastatic sites, more M2 TAMs were present compared to primary tumor sites. We also discovered that neoadjuvant chemotherapy tends to increase these M2 and that bevacizumab does not alter this effect. Visual inspection revealed a striking co-location of intratumoral blood vessels and TAM, an increase of M2 with increase blood vessel abnormality (measured by blood vessel width), and an increase of this abnormality by neoadjuvant chemotherapy and bevacizumab. 

Our findings at diagnosis where M2 TAMs are increased at metastatic sites is in line with previous literature data showing that M2 increase the invasiveness of ovarian cancer [24] and that TAM behavior, number, and composition differ according to the tumor area (demonstrated in different solid tumors, but not yet in ovarian cancer [25]). Although often treated similarly, low-grade tumors respond differently to chemotherapy compared to high-grade tumors [26], differ genetically [27], and have a different immune biology. The group of Yang et al. demonstrated, based on the retrospective analysis of micro-array datasets, that the gene signature of immune cells is nearly opposite in high-grade serous ovarian cancer compared to low-grade serous ovarian cancer [28]. The expression of B7-H4 (regulating T-cell immunity) was significantly reduced in low-grade serous ovarian cancer compared to high-grade serous ovarian cancer [29]. Our group recently demonstrated a reduced innate immune suppression in blood samples of low-grade ovarian cancer patients compared to high-grade ovarian cancer [11]. The current pilot study adds a high M1/M2 ratio at the protein level to the immune knowledge of low-grade ovarian tumors. This less immune suppressive landscape might be an advantage for the success rate of immunotherapy in this type of tumor. However, few clinical studies addressed this niche (e.g., NCT02923934 focusing on immune checkpoint inhibitor therapy in rare cancers, including low-grade ovarian cancers). 

The evaluation of tumor biopsies after neoadjuvant chemotherapy revealed an increase in vessel width, TAM, and M2. We could demonstrate that blood vessel width is correlated with M2 presence, but the cause and the consequence are not so clear. In vitro experiments showed that paclitaxel-carboplatin increases CCL2 (chemokine (C-C) motif ligand 2) [30]. CCL2 is known to attract macrophages. In murine ovarian cancer experiments, inhibition of CCL2 increased the response to carboplatin-paclitaxel [31]. However, the immune system is much more than only macrophages and, to evaluate the immune-stimulating or immune-suppressive effects of carboplatin-paclitaxel in ovarian cancer, the whole immune system has to be taken into account. Our group highlighted in this regard that immune suppression measured in ascites is reduced after neoadjuvant chemotherapy [32], and that carboplatin-paclitaxel can render the cytokine profile in serum of ovarian cancer patients less immunosuppressive, through a decrease in interleukin-10, VEGF, transforming growth factor-β, and arginase [33]. Moreover, taking into account that the immune system is most likely different in different metastatic biopsies of ovarian cancer patients [34,35,36], we have to be cautious in interpreting data after treatment based on one tumor biopsy. 

Furthermore, the data on bevacizumab have to be considered with this in mind. Anti-VEGF therapy, which was proven beneficial in ovarian cancer [20], is indeed known to modify the immune contexture [37] and is expected to reduce the amount of M2 macrophages. On the other hand, there is evidence that the presence of M2 macrophages can be an indication of resistance to anti-VEGF therapy [22,38,39]. It inhibits angiogenesis but can also promote the recruitment of TAM, immature monocytes, and other vascular modulators to the tumor site [39]. Furthermore, M2 TAMs are known to produce a wide variety of proangiogenic factors such as VEGF to further enhance tumor vascularization, eventually resulting in therapy resistance [40,41]. Moreover, MMP-9 (matrix metalloproteinase 9) secreted by macrophages can increase the bioavailability of VEGF to its receptor [42,43]. Therefore, our observation that bevacizumab in combination with carboplatin-paclitaxel increases the blood vessel diameter and the amount of TAM and M2 in comparison to chemotherapy alone might (albeit having only one biopsy) be relevant to take into account in combinatorial immunotherapy design (already ongoing in, for example, the IMagyn050 (NCT03038100) and DUO-O (NCT03737643) studies). Increase in immunosuppression can alter the response to immunotherapy.

We did not report on the correlation of macrophages and survival, notwithstanding the fact that we have survival data on all patients. The number of patients was too small to perform reliable statistics. Nevertheless, in the literature, there is compelling evidence that M2 TAMs worsen the prognosis of ovarian cancer patients (meta-analysis of Yuang et al. in nine studies on 794 patients) [44].

Of course, this study had limitations, such as the small sample size, the lack of several metastatic biopsies of one patient to compare [34,35,36], and the substantial variation between macrophage counts in biopsies under treatment. Another important limitation of this study was the use of a single marker to define the M1 and M2 TAM subsets, MHCII and MRC1, respectively. Macrophages are a very diverse set of cells that constantly shift their functional state and consequently also display altered expression levels of markers [12]. Therefore, multiple expression markers will be required in future studies to be able to distinguish more subsets of macrophages such as M2a, M2b, and M2c subtypes [45]. However, our pilot study was designed to detect possible trends worth further investigating in the future and not to perform a full characterization of different TAM subsets.

The most important finding of this pilot project is without any doubt the clear discrepancy between high-grade and low-grade ovarian cancer, with a less immune suppressive profile in low-grade ovarian cancer. This might open up possibilities for altered therapeutic management in this less common subtype of ovarian cancer. 

## Figures and Tables

**Table 1:** Patient characteristics (*n* = 24).

Characteristic		Result (Absolute/Percentage)
Age (median (years), range)		63 (47–85)
Histological subtype	Serous	21 (88)
	Endometroid	1 (4)
	Clear-cell carcinoma	1 (4)
	Carcinosarcoma	1 (4)
Differentiation grade	High-grade	21 (88)
	Low-grade	3 (12)
Stage	I	3 (12)
	III	11 (46)
	IV	10 (42)
Surgical treatment	Upfront primary debulking	11 (46)
	Interval debulking surgery	13 (54)
Chemotherapy treatment	Carboplatin-paclitaxel	10 (42)
	Carboplatin monotherapy	2 (8)
	Carboplatin-paclitaxel + bevacizumab	10 (42)
	Other (letrozole, interruption of chemo because of toxicities)	2 (8)
Outcome	No evidence of disease	5 (21)
	Alive with evidence of disease	8 (33)
	Death of disease	11 (46)
Progression-free survival	Median (months), range	21 (6–60)
Bioptic site	Total amount of biopsies	30
	Primary tumor at diagnosis	11 (37)
	Metastasis at diagnosis	6 (20)
	Primary tumor at interval debulking	9 (30)
	Metastasis at interval debulking	4 (13)
	Paired samples (primary tumor + metastasis)	5 (17)
	Paired samples (before and after neoadjuvant chemotherapy)	(3)

## Supplementary Materials

The following are available online at https://www.mdpi.com/2073-4409/9/2/305/s1, Figure S1: Bulk analysis of primary and metastatic tumor site biopsies.
