# Supplementary material for: Opposite Macrophage Polarization in Different Subsets of Ovarian Cancer: Observation from a Pilot Study
Source: Cells. 2020 Jan 27;9(2):305. doi: 10.3390/cells9020305 (PMC7072171; doi:10.3390/cells9020305)
Supplement: Supplementary file 1 [file cells-09-00305-s001.pdf]

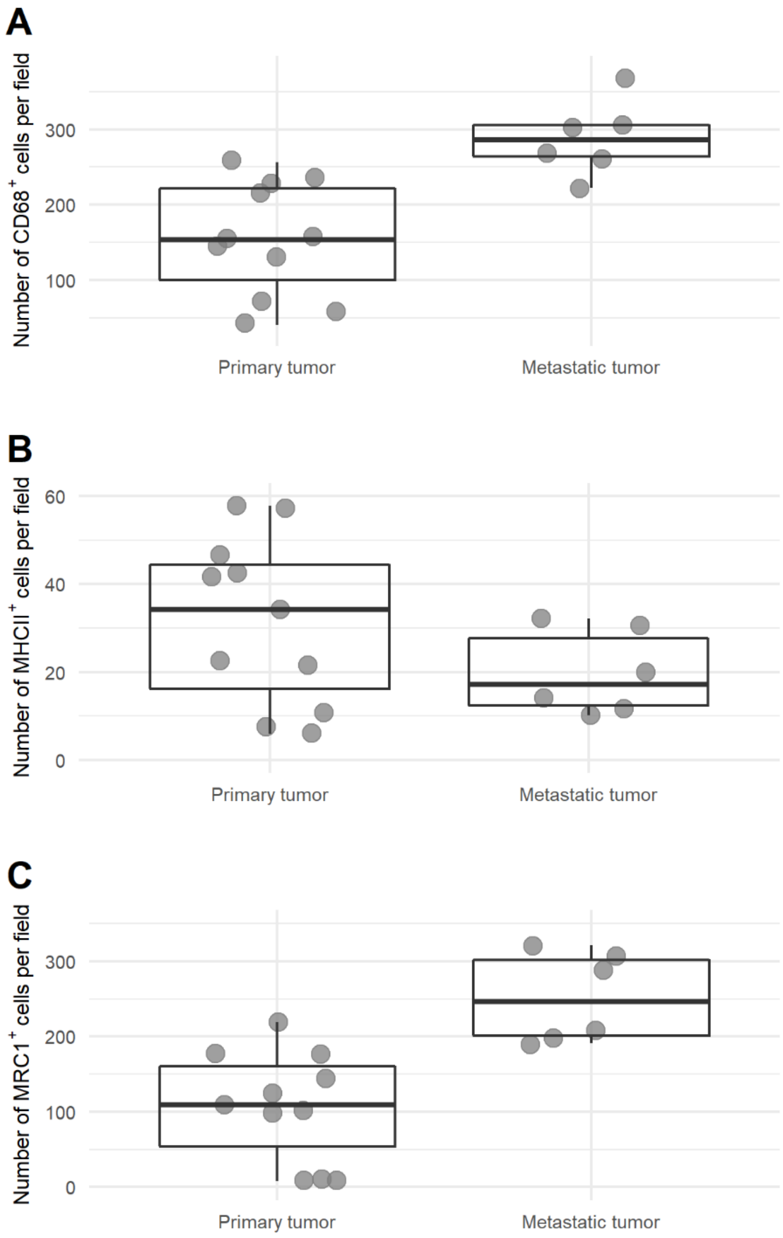

Supplementary Figure 1. Bulk analysis of primary and metastatic tumor site biopsies stained for number of cells being (a) $CD68^+$ (b) $MHCII^+$ (c) $MRC1^+$.
